# Analysis of Relative Abundance Distribution and Environmental Differences for Blue Mackerel (*Scomber australasicus*) and Chub Mackerel (*Scomber japonicus*) on the High Seas of the North Pacific Ocean

**DOI:** 10.3390/ani15192822

**Published:** 2025-09-27

**Authors:** Heng Zhang, Hanji Zhu, Famou Zhang, Sisi Huang, Jianhua Wang, Delong Xiang, Yang Li, Yuyan Sun

**Affiliations:** 1 Key Laboratory of Oceanic and Polar Fisheries, Ministry of Agriculture and Rural Affairs, P.R. China, East China Sea Fisheries Research Institute, Chinese Academy of Fishery Sciences, Shanghai 200090, China; zhangziqian0601@163.com (H.Z.); mikey0987@163.com (H.Z.); 13276209271@163.com (F.Z.); huangsisi3254@163.com (S.H.); wjh20001231@163.com (J.W.); xdl17852167218@163.com (D.X.); 2 College of Marine Living Resource Sciences and Management, Shanghai Ocean University, Shanghai 201306, China; 3 Wenchang Innovation Research Center of East China Sea Fisheries Research Institute, Chinese Academy of Fishery Sciences, Wenchang 571343, China; 4 College of Navigation and Ship Engineering, Dalian Ocean University, Dalian 116023, China; 5 School of Geography and Ocean Science, Nanjing University, Nanjing 210023, China

**Keywords:** Blue Mackerel, chub mackerel, co-occurring species, Zero-One Inflated Beta Model (ZOIBM), resource abundance distribution

## Abstract

The accurate assessment and management of Blue Mackerel (***Scomber australasicus***) and Chub Mackerel (***Scomber japonicus***) resources in the high seas of the Northwest Pacific are constrained by the persistent issue of data misreporting in catch records, which arises from their high morphological similarity. This study integrates fishery logbooks and field sampling data from Chinese purse seine fleets (2014–2023), along with key oceanographic factors—six of which were finally selected after correlation analysis. We introduce, for the first time, a Zero-One Inflated Beta Model (ZOIBM) to analyze the spatiotemporal distribution of the relative abundance of these two mackerel species. Furthermore, a Generalized Additive Model (GAM) was employed to reveal the environmental mechanisms driving their niche differentiation. The results show that the ZOIBM demonstrates excellent performance (R^2^ = 0.63, RMSE = 0.305), effectively quantifying the proportional composition of the two species in mixed catches. Spatially, high-abundance areas of Blue Mackerel were concentrated within 35–44° N, 145–160° E, with its proportion decreasing at higher latitudes. In contrast, Chub Mackerel exhibited an opposite latitudinal pattern, with its high-abundance areas covering a broader latitudinal range (35–47.5° N). The analysis of environmental drivers indicated that SST was the most critical factor for differentiation, while Chla and VO further amplified the divergence in resource utilization strategies between the species. From 2014 to 2023, the distribution centroids of both mackerel species showed significant northward and eastward shifts, and their spatial overlap has been continuously increasing. This research provides a methodological reference for the fine-scale assessment of co-occurring fish resources and offers a scientific basis for the sustainable management of the North Pacific mackerel fishery.

## Simple Summary

Blue Mackerel and Chub Mackerel are two economically important fish in the North Pacific Ocean that look very similar. Because they are difficult to tell apart, fishermen often report them together in catch records. This makes it hard for scientists to accurately assess the health of each species’ population. This study used fishing data from 2014 to 2023 and advanced computer models to distinguish between the two species in mixed catches. We successfully mapped where each species is most abundant. We found that Blue Mackerel prefer warmer, southern waters, while Chub Mackerel are more common in cooler, northern areas. Key environmental factors like sea surface temperature (SST) and Chlorophyll-a concentration (Chla) were found to drive these different distributions. Over the past decade, we observed that both species are moving northward and eastward, likely in response to climate change. As their habitats shift, their populations are overlapping more, which could lead to increased competition. Our findings provide a new method for accurately assessing these species and offer a scientific basis for better, more sustainable fishery management in the North Pacific.

## 1. Introduction

The North Pacific Ocean, spanning from subtropical to subarctic regions, constitutes one of the world’s largest marine ecosystems due to its complex and diverse oceanographic environment [1]. The confluence of the cold Oyashio Current and the warm Kuroshio Current enriches the region with abundant nutrients, establishing it as one of the most important and productive fishing grounds globally [2]. The rich biological resources of this area not only drive the development of the regional blue economy but also contribute significantly to global marine biodiversity and ecological balance. Its unique geographical location and complex marine environment provide ideal habitats and breeding grounds for a multitude of fish, invertebrates, and other marine life. Furthermore, the region has a long history of fishing activities, targeting species of high economic value [3,4,5]. However, many fishery resources in this area are facing increasingly severe challenges due to the impacts of global climate change (e.g., rising sea temperatures, ocean acidification), habitat degradation (e.g., industrial pollution), and maritime activities such as shipping (e.g., noise pollution, oil spills) [6,7,8].

**Figure 1:**
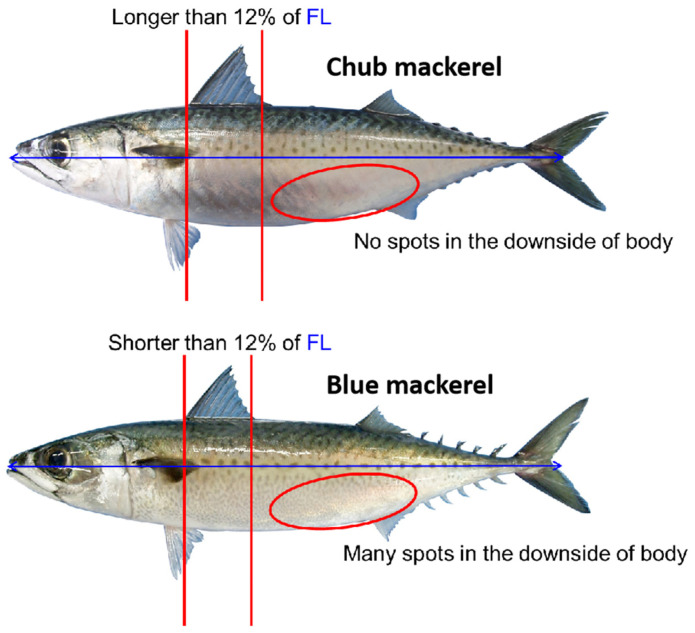
Morphological differences between chub mackerel (***Scomber japonicus***) and blue mackerel (***Scomber australasicus***) (The image is provided by the NPFC Technical Working Group on Chub Mackerel Stock Assessment; FL means fork length).

Mackerels, primarily comprising Blue Mackerel (***Scomber australasicus***) and Chub Mackerel (***Scomber japonicus***), are important commercial fish species in the North Pacific Ocean [9]. As key small pelagic species, they fulfill dual ecological roles as both predators and prey, contributing significantly to the dynamics of the marine food web. They also have potential interspecific competition due to overlapping distributions (Northwest Pacific) and similar niches—both are small pelagics feeding on plankton and small fish [10]. Their pivotal position in regulating the population dynamics of both lower and higher trophic levels underscores their ecological importance [11,12]. For many years, fishery logbooks have been an indispensable source of data for documenting mackerel fishing activities. The accuracy and timeliness of these records are crucial for understanding the resource status and spatial distribution patterns of mackerel fisheries, forming the basis for rational planning and scientific management of these resources [3,4]. However, a long-standing and challenging problem exists within mackerel fishery logbook data: the inability to differentiate catch quantities by species in real-time at sea. Due to the high morphological similarity and equivalent commercial value of Blue and Chub Mackerel, catches of these two species are often aggregated and recorded together by fishers in practice, without species-specific separation (Figure 1). This practice of aggregated reporting overlooks the biological and ecological differences between the two, hindering accurate stock assessments and undermining the effectiveness of current resource quota management policies for these two species, which are priority stocks under the jurisdiction of the North Pacific Fisheries Commission (NPFC) [13]. Therefore, accurately distinguishing between these two co-occurring species is critical for a more precise determination of their true abundance and spatial distribution, particularly against the backdrop of a marine ecosystem continuously changing under the influence of climate change.

Against the backdrop of rapid advancements in computational modeling and remote sensing technology, new opportunities have emerged for identifying the resources of co-occurring fish species [14], for instance, successfully delineated the core distribution areas of lobster populations off the coast of Australia using a Boosted Regression Trees (BRT) approach combined with high-resolution environmental data, showcasing the potential of data-driven models in complex species discrimination. This provides a technical pathway for exploring the spatial differentiation and resource modeling of Blue and Chub Mackerel. Statistical modeling methods, in particular, can effectively handle issues such as high-dimensional environmental variables and incomplete sample data by constructing environment-distribution relationship models to accurately predict potential species distributions. Meanwhile, the high spatiotemporal resolution of remote sensing data provides robust support for niche differentiation analysis. This study introduces, for the first time, the Zero-One Inflated Beta Model (ZOIBM) to model the proportion of mackerels in mixed-catch data, aiming to distinguish the resource abundance and distribution of Blue and Chub Mackerel. Compared to traditional classification models like BRT, which can only handle binary presence-absence data, the ZOIBM can quantify the relative proportions of the two co-occurring species at a given location, overcoming the binary “either-or” constraint. By simultaneously handling continuous proportional data and the boundary values of 0 and 1, this model offers a new paradigm, both theoretically and empirically, for estimating the resources of co-occurring species [15,16,17].

This study focuses on two core scientific questions: (1) Characterizing the spatiotemporal distribution patterns and dynamic evolution of the two mackerel species using the Zero-One Inflated Beta Model (ZOIBM); and (2) Identifying the key combinations of environmental factors that drive their niche differentiation.

Notably, the ZOIBM also provides an innovative solution to address the limitations of existing fishery data. As a model-based data calibration tool, the integration of this calibration method with fishermen’s fish species identification training can effectively improve the accuracy of real-time catch reporting. This not only offers more reliable quantitative support for the management of mackerel resources in the Northwest Pacific but also provides a methodological reference for distribution modeling and climate response analysis of co-occurring species.

## 2. Materials and Methods

### 2.1. Data Sources

#### 2.1.1. Fishery Data Sources

The first component comprises logbook data from Chinese commercial purse seiners operating in the high seas of the Northwest Pacific (2014–2023), which includes mixed catch records of the two mackerel species. The data encompasses georeferenced locations (latitude/longitude), time-series information (year, month, day, hour), haul counts, and daily catch (kg). Recorded daily by captains and submitted for reporting, this data is subject to dual supervision by the North Pacific Fisheries Commission (NPFC) and the China National Fisheries Association, thus boasting high accuracy. The second component consists of field sampling data for mackerels in the region. This data was collected through multiple random monthly samplings conducted by several Northwest Pacific purse seiners in fishing grounds. Morphological identification and enumeration were employed to determine the species proportion in the total catch at each sampling site. Serving as input for model training, this data forms the basis for subsequent spatial distribution analysis.

#### 2.1.2. Acquisition and Selection of Environmental Data

The spatiotemporal dynamics of mackerel resources on the high seas of the Northwest Pacific are regulated by multidimensional oceanographic factors. Therefore, the scientific selection of environmental variables is a critical prerequisite for ensuring predictive accuracy. Previous studies have indicated that factors such as Sea Surface Temperature (SST), Chlorophyll-a concentration (Chla), Sea Surface Salinity (SSS), Sea Surface Height (SSH), Northward sea water velocity (VO), Eastward sea water velocity (UO), and Mixed Layer Depth (MLD) significantly influence mackerel distribution [18]. SSH reflects dynamic oceanographic processes like ocean currents and eddies, and its variations are vital to both Blue Mackerel and Chub Mackerel distribution—by modulating water mass transport to affect population dispersal and habitat access, and by driving prey aggregation via eddy-related anomalies. Among these, Blue Mackerel exhibits a distinct preference for high-temperature and high-salinity environments [19], resulting in a differentiated temperature-salinity niche compared to Chub Mackerel. Chla, a core indicator of marine primary productivity, is directly associated with the forage base for small pelagic fish; variations in its concentration affect phytoplankton biomass and the efficiency of energy transfer through the food chain [20]. VO and UO critically influence the spatial expansion of mackerel schools by regulating forage distribution and population dispersal boundaries—the schooling behavior of these species makes their distribution range highly coupled with the ocean current transport capacity [21]. From the perspective of physiological and ecological mechanisms, SST not only regulates the metabolic rates of mackerel but also constrains their migration routes and suitable habitat range by affecting enzyme activity and osmoregulation processes [9,22,23]. Variations in SSS interfere with the electrolyte balance in fish; anomalous salinity environments can lead to reduced reproductive capacity or impaired growth and development [19,24]. MLD, by influencing the efficiency of vertical mixing in the water column, alters the spatial distribution of nutrients and dissolved oxygen, thereby reshaping the three-dimensional habitat of the fish [25,26]. Based on this theoretical foundation, this study selected a total of seven environmental factors—SST, VO, UO, Chla, SSH, SSS, and MLD—as modeling variables (Table 1). The corresponding data sources and spatiotemporal resolutions are as follows:

#### 2.1.3. Environmental Data Processing

The spatiotemporal consistency and independence of environmental variables are fundamental to constructing high-precision niche models. In this study, data preprocessing was conducted using a Python 3.10 script, with the following specific steps: First, given that the spatial resolution of the original daily Chla data differed from that of the other environmental datasets, potentially compromising the accuracy of subsequent analyses and modeling, the daily Chla data were resampled to a uniform resolution (0.083° × 0.083°) using the rasterio library to align the spatial scales. After resampling the Chla data, the daily environmental raster data from 2014 to 2023 were matched with the geodetic coordinates (latitude and longitude) of the fishery logbooks based on the nearest neighbor method. This process extracted the corresponding SST, VO, UO, Chla, SSH, SSS, and MLD values for each catch record.

**Figure 2:**
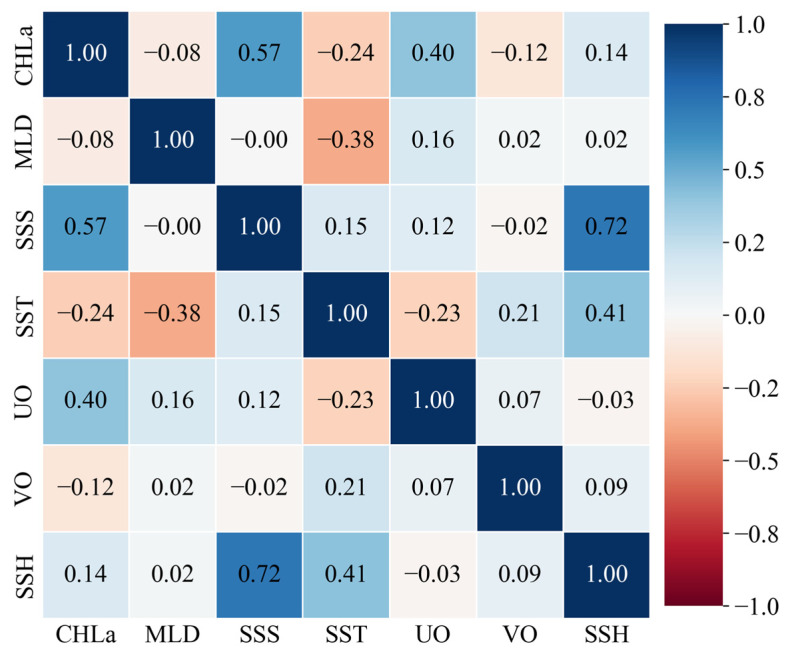
Heat map of Pearson correlation coefficients of seven environmental factors.

To prevent multicollinearity from affecting the stability of the model, a Pearson correlation test was performed on the seven candidate environmental variables (Figure 2). According to standard statistical criteria, a Pearson correlation coefficient (r) with an absolute value below 0.7 (|r| < 0.7) is generally considered to indicate a lack of significant correlation between variables [27]. In our analysis, the correlation coefficient between SSS and SSH was 0.72, demonstrating a significant positive correlation. Considering the critical role of SSS in differentiating the ecological niches of Blue Mackerel and Chub Mackerel [19], SSH was excluded, and the final set of input variables for the model included SST, UO, VO, Chla, MLD, and SSS. This variable selection strategy effectively reduced dimensional redundancy and ensured the relative independence among the environmental factors, thereby providing a robust foundation for the subsequent ZOIBM.

### 2.2. Construction of the ZOIBM

This study constructed a ZOIBM within a Bayesian framework using the brms package in R (version 4.2.3) to characterize the proportional composition of the two mackerel species in mixed catch records. This model combines a continuous Beta distribution with a discrete mechanism for the extreme values of 0 and 1, enabling it to effectively handle response variables that lie within the (0, 1) interval but frequently include exact 0 s and 1 s. This makes it highly suitable for modeling the proportional data of co-occurring species [28,29,30]. The Beta distribution was fitted to the daily catch proportion of Blue Mackerel and Chub Mackerel (2014–2023), while the zero- and one-inflation components captured the boundary values; all MCMC sampling was conducted directly on this dataset. Four Markov Chain Monte Carlo (MCMC) chains were run for 4000 iterations each, with the first 2000 iterations discarded as burn-in to eliminate initial value bias. To enhance sampling efficiency and model stability, adapt_delta was set to 0.99 and max_treedepth to 15. A spatial random effect term was included to control for spatial autocorrelation, and a weakly informative Laplace prior was set for the regression coefficients to mitigate overfitting. Model generalization was assessed using 5-fold cross-validation via the k-fold function in brms. The final model thus comprises three components: the Beta distribution modeling proportions within (0, 1), and the zero- and one-inflation components modeling the probability of exact 0 or 1, reflecting species composition at different fishing locations. Environmental covariates were included in both the zero and one-inflation components as well as the Beta component, ensuring that predictors consistently explain both extreme and non-extreme proportions.

### 2.3. ZOIBM Evaluation Metrics

To systematically validate the reliability of the ZOIBM, this study established a multi-dimensional evaluation framework to assess model performance in terms of convergence, goodness-of-fit, and predictive consistency. By integrating Bayesian statistical diagnostics with classic goodness-of-fit metrics, we ensured the reliability of the model in parameter estimation, data fitting, and predictive generalization.

#### 2.3.1. MCMC Convergence Diagnostics

The convergence status of the Markov chains was assessed using the Gelman-Rubin statistic (R-hat). This metric quantifies the stability of the MCMC sampling by comparing the variance between chains to the variance within each chain. When the R-hat values for all parameters are below 1.05, the chains are considered to have mixed sufficiently, and the posterior distribution estimates are deemed stable [31,32].

#### 2.3.2. Posterior Predictive Checks

Posterior predictive checks were used to verify the consistency between the model’s assumptions and the data-generating process. A total of 100 simulated datasets (*yrep*) were randomly drawn from the model’s posterior distribution and compared against the observed data (*y*) to assess their distributional overlap. If the model’s assumptions are valid, the *yrep* datasets should be able to replicate the key statistical features of *y* (e.g., distribution of extreme values, fluctuation patterns, and quantile characteristics). Significant distributional discrepancies would suggest potential structural misspecification or inadequate assumptions in the model [33,34].

#### 2.3.3. Quantification of Goodness-of-Fit

The model’s ability to explain the training data was quantified using the following metrics: R^2^ (Coefficient of Determination): Represents the proportion of variance in the data explained by the model. It ranges from 0 to 1, with higher values indicating a better fit. MAE (Mean Absolute Error) and RMSE (Root Mean Square Error): These metrics measure the average absolute deviation and the root mean square deviation between predicted and observed values, respectively. Smaller values for both indicate higher prediction accuracy [35]. The specific formulas are as follows:

(1) $$\mathrm{RMSE}\;=\;\sqrt{\frac{1}{m\;}\sum_{i=1}^{m\;}{\left(y_{i}-{ŷ}_{i}\right)}^{2}}$$

(2) $$\mathrm{MAE}=\frac{1}{m\;}\sum_{i=1}^{m\;}\left|y_{i}-{ŷ}_{i}\right|$$

(3) $$\;\;R^{2\;}=1-\frac{\sum_{i=1}^{m\;}{\left(y_{i}-{ŷ}_{i}\right)}^{2}}{\sum_{i-1}^{m\;}{\left(y_{i}-{\bar{y}}_{i}\right)}^{2}}$$

where $y_{i}$ is the observed value for the i-th sample, ${ŷ}_{i}$ is the predicted value for the i-th sample, and m is the total number of samples.

### 2.4. GAM Construction

To analyze the differential effects of environmental factors on the abundance distribution of Blue Mackerel and Chub Mackerel, this study employed a Generalized Additive Model (GAM) to model the non-linear relationships. As a non-parametric extension of Generalized Linear Models (GLMs), a GAM utilizes spline functions to smoothly fit complex non-linear relationships between explanatory variables and a response variable. This approach has been widely applied in the field of ecological niche analysis for fishery resources [36,37]. During the data preprocessing stage, to address the issue of zero values in the catch (which interfere with logarithmic transformation), a log-plus-one transformation was applied to the data. Specifically, the response variable was formulated as ln(catch + 1) to meet the model’s distributional assumptions. The GAM formulated in this study is specified as follows:

(4) $$\log\mathrm{Catch}+1\sim S\left(\mathrm{SST}\right)+S\left(\mathrm{Chla}\right)+S\left(\mathrm{UO}\right)+S\left(\mathrm{VO}\right)+S\left(\mathrm{MLD}\right)+S\left(\mathrm{SSS}\right)+\varepsilon$$

where Catch denotes the catch per vessel per set of the two mackerel species calculated by the Zero-Inflated Bayesian Model (ZOIBM), with the unit of “catch per set”; *S* represents a spline smoothing function, which can be used for univariate smoothing and isotropic multivariate smoothing; and ε is the error term. The model was constructed using the mgcv package in RStudio (version 4.2.3). From the simulation results, the optimal environmental range for the target species was defined as the interval where the 95% confidence bands around the fitted curve were narrowest [38].

### 2.5. Annual Variation in the Centroid for Blue Mackerel and Chub Mackerel

Investigating the spatiotemporal dynamics of the distribution of Blue Mackerel and Chub Mackerel from 2014 to 2023 is of significant scientific value for analyzing migration strategies, quantifying the ecological effects of climate change, and optimizing fishery resource management. In this study, the spatial aggregation patterns of the two mackerel species were quantified by calculating the annual centroid based on their catch proportions. The specific method, using latitude and longitude coordinates, is as follows:

(5) $${\mathrm{weighted}}_{\mathrm{lon}}=\sum_{i=1}^{n}\frac{\left({\mathrm{longitude}}_{i}\times {\mathrm{proportion}}_{i}\right)}{\sum_{i=1}^{n}{\mathrm{proportion}}_{i}}$$

(6) $${\mathrm{weighted}}_{\mathrm{lat}}=\sum_{i=1}^{n}\frac{\left({\mathrm{latitude}}_{i}\times {\mathrm{proportion}}_{i}\right)}{\sum_{i=1}^{n}{\mathrm{proportion}}_{i}}$$

where $\;{\mathrm{weighted}}_{\mathrm{lon}}$ and ${\mathrm{weighted}}_{\mathrm{lat}}$ represent the longitude and latitude of the centroid for the catch proportion of a given mackerel species; ${\mathrm{longitude}}_{i}$ is the longitude of the i-th data point; ${\mathrm{latitude}}_{i}$ is the latitude of the i-th data point; ${\mathrm{proportion}}_{i}$ is the proportion of that species at the i-th data point; and n is the total number of data points.

## 3. Results

### 3.1. ZOIBM Accuracy Evaluation

#### 3.1.1. Convergence and Parameter Estimation

The results for the Gelman-Rubin statistic (R-hat) of the model parameters (Table 2) show that all R-hat values ranged from 0.9996 to 1.0047, well below the critical threshold of 1.05. This metric indicates that the four Markov chains fully converged, resulting in stable posterior distributions. This outcome mitigates the risk of biased parameter estimates and confirms the reliability and robustness of the model parameters.

#### 3.1.2. Posterior Predictive Assessment of ZOIBM Adequacy

**Figure 3:**
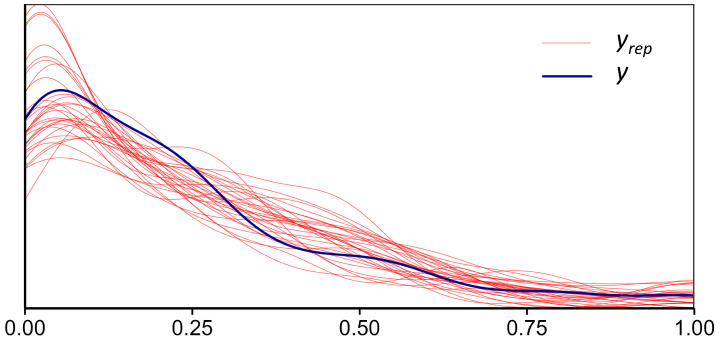
Posterior predictive check for the fitted ZOIBM. The observed data (*y*) represent the daily proportion of each mackerel species in the catch (range: 0–1, dimensionless). The simulated data (*yrep*) are 100 posterior predictive replicates drawn from the zero-one inflated beta distribution. The x-axis denotes proportion, and the y-axis denotes probability density (unitless).

One hundred sets of simulated data (*yrep*) were randomly drawn from the model’s posterior distribution and their distributions were compared with that of the actual observed data (*y*). The results show a high degree of congruence between the simulated and observed distributions (Figure 3), accurately reproducing the distributional characteristics, including the aggregation at the extremes (0 and 1) and the pattern of intermediate proportions. This validates the strong fit between the model’s assumptions and the underlying data structure.

#### 3.1.3. Goodness-of-Fit Assessment

The generalization performance of the model was evaluated using block cross-validation. The results indicated that, on the test set, the model achieved a MAE of 0.298, reflecting an average deviation of 29.8% between predicted and observed values. RMSE was 0.305, demonstrating a high level of predictive accuracy. Notably, the model’s Coefficient of Determination (R^2^) reached 0.63, signifying that the model could explain 63% of the variance in the test set data. Collectively, these metrics confirm the effectiveness of the ZOIBM in handling proportional data on the [0, 1] interval.

### 3.2. Effects of Environmental Factors on the Relative Abundance Distribution of the Two Mackerel Species

Generalized Additive Models (GAMs) were constructed separately for the yields of Blue Mackerel and Chub Mackerel to fit environmental factors. The results showed that the *p*-values of all smooth terms were less than 0.001, indicating the models were statistically significant and robust. Both the effective degrees of freedom (edf) and reference degrees of freedom (Ref.df) were greater than 1 and close to each other, suggesting a strong non-linear relationship between the variables and the response variable, and that the models could fully capture the complex patterns in the data. Regarding the overall model performance, the GAM for Blue Mackerel; achieved an R^2^ of 0.467 and a deviance explained of 0.473, while the GAM for Chub Mackerel had an R^2^ of 0.426 and a deviance explained of 0.433. These results further confirm the reliability of the models (Table 3).

**Figure 4:**
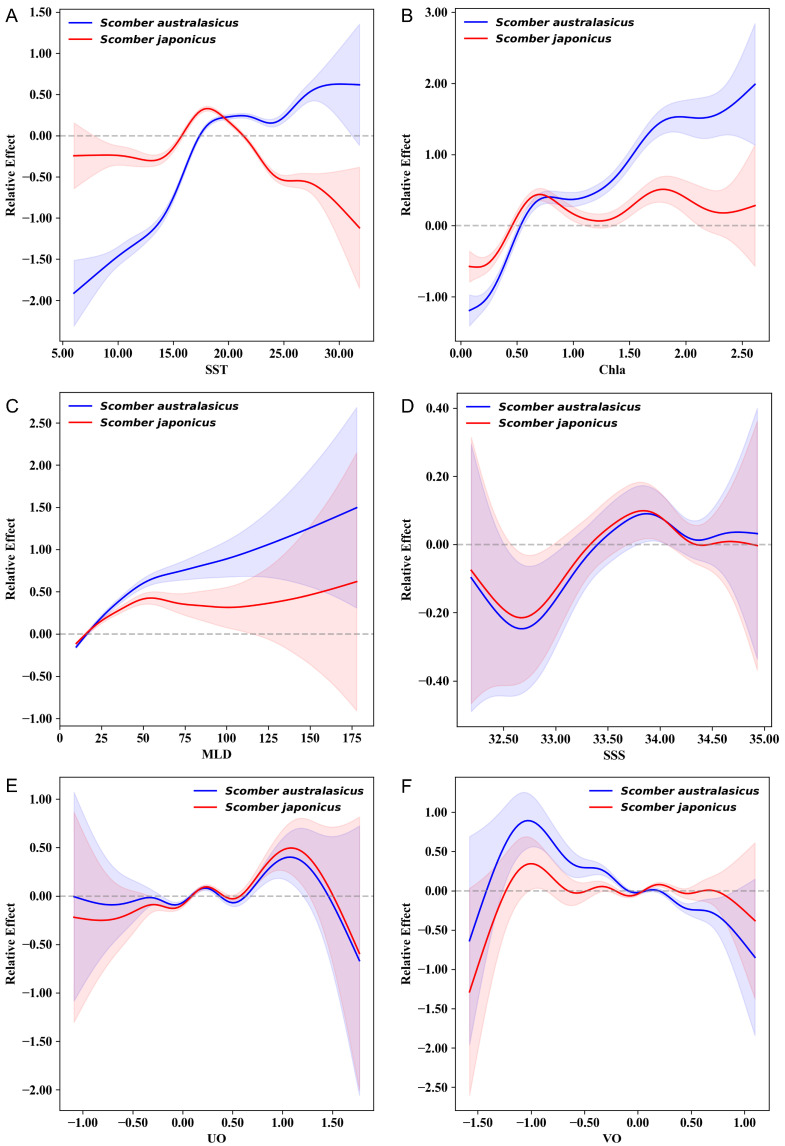
Relationships between environmental factors and the relative abundance of two mackerel species. (**A**) SST, (**B**) Chla, (**C**) MLD, (**D**) SSS, (**E**) VO, (**F**) UO. Blue and red lines (with shaded confidence intervals) represent Blue Mackerel (***Scomber australasicus***) and Chub Mackerel (***Scomber japonicus***), respectively.

The analysis of the smoothing terms from the GAM revealed the differentiated driving mechanisms of key environmental factors on the abundance distribution of Blue Mackerel and Chub Mackerel. Figure 4A indicates that there is a significant difference in the response of Chub Mackerel and Blue Mackerel to SST. The relative abundance of Chub Mackerel showed a unimodal response, reaching its peak within the approximate range of 17–19 °C before declining rapidly, suggesting a narrow optimal habitat temperature and a higher sensitivity to warm water. In contrast, Blue Mackerel exhibited a monotonically increasing response to SST, maintaining a high probability of occurrence even in the warmest areas of the study region (approx. 23 °C), which demonstrates its stronger high-temperature tolerance. Figure 4B shows that Chla had a non-linear effect on both species, with marked differences. As Chla increased, the partial effect on Blue Mackerel first rose, then stabilized, before increasing rapidly again. The partial effect on Chub Mackerel showed only minor fluctuations with changes in Chla, indicating that this species is relatively less influenced by Chla. As shown in Figure 4C, both mackerel species exhibited a positive correlation with MLD, but with slightly different response intensities. The probability of occurrence for Chub Mackerel showed a more pronounced and continuous upward trend as MLD increased. Figure 4D reveals that the response of both species to SSS was broadly similar, showing a unimodal pattern that was high in the middle and low at the extremes. Chub Mackerel reached its maximum probability of occurrence at approximately 33.9‰, whereas Blue Mackerel peaked under slightly higher salinity conditions near 34.0‰. Their optimal salinity ranges were highly overlapping. Figure 4E indicates that the responses to UO were nearly identical for both species. Both peaked at a velocity of 0 m/s and subsequently showed a symmetrical decreasing trend, suggesting that UO plays a weak role in their niche differentiation. As depicted in Figure 4F, the response of both species to VO showed a downward trend, but the decline was more pronounced for Blue Mackerel.

### 3.3. Relative Abundance Distribution of Blue Mackerel and Chub Mackerel

**Figure 5:**
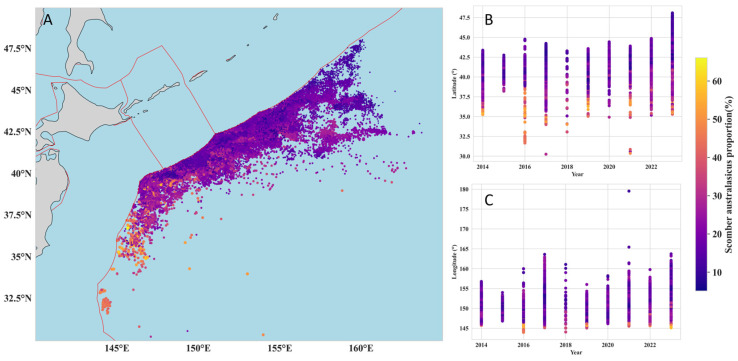
Spatiotemporal distribution of the proportion of Blue Mackerel (***Scomber australasicus***) in total catch from 2014 to 2023. (**A**) Geographic distribution of catch proportions. The red lines indicate the boundaries of the Exclusive Economic Zone (EEZ). (**B**) Proportional distribution by latitude and year. (**C**) Proportional distribution by longitude and year.

To investigate the changes in the distribution patterns of Blue Mackerel and Chub Mackerel, this study mapped the spatiotemporal distribution of the proportion of each species in the total catch from 2014 to 2023 (Figure 5).

**Figure 6:**
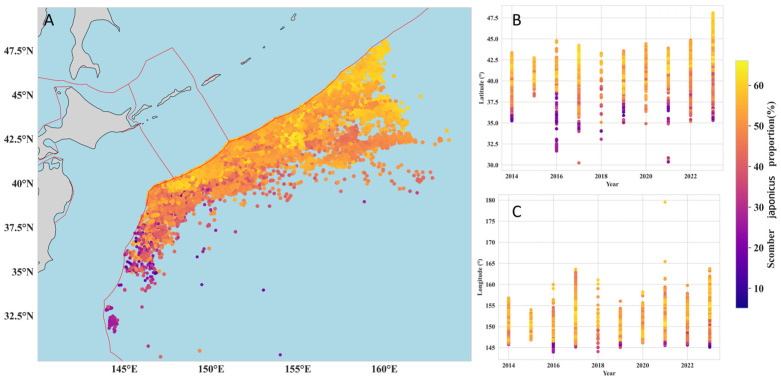
Spatiotemporal distribution of the proportion of Chub Mackerel (***Scomber japonicus***) in total catch from 2014 to 2023. (**A**) Geographic distribution of catch proportions. The red lines indicate the boundaries of the Exclusive Economic Zone (EEZ). (**B**) Proportional distribution by latitude and year. (**C**) Proportional distribution by longitude and year.

From a spatial perspective (Figure 5A), Blue Mackerel exhibited a distribution pattern of regional aggregation on the high seas of the Northwest Pacific. Its high-proportion distribution areas were concentrated in the region of approximately 35–44° N, 145–160° E. In the high-latitude regions north of 45°N, the proportion of Blue Mackerel was consistently below 10%. The latitude-year distribution plot (Figure 5B) shows that while the proportion of Blue Mackerel fluctuated across latitudes from year to year, it displayed an overall trend of “decreasing proportion with increasing latitude”. Along the longitudinal dimension, the proportion of Blue Mackerel showed no significant pattern, except in the 145–146° E band, where its proportion was significantly higher than in other longitudes (Figure 5C). In contrast, the spatial distribution of Chub Mackerel revealed an opposite latitudinal pattern (Figure 6A), with its proportion increasing significantly as latitude increased (Figure 6B). The high-proportion areas for Chub Mackerel were concentrated at approximately 35–47.5° N (Figure 6B), and the latitudinal range covered by these high proportions was significantly wider than that of Blue Mackerel. Longitudinally, the proportion of Chub Mackerel did not show a clear pattern, except in the 145–146° E band, where its proportion was significantly lower than in other longitudes.

### 3.4. Dynamics of the Centroid of Abundance for Blue Mackerel and Chub Mackerel

**Figure 7:**
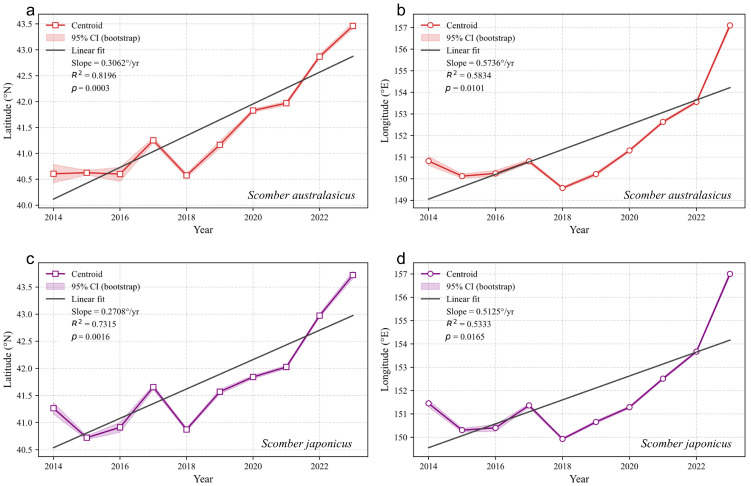
The dynamics of distribution centroids for Blue Mackerel (***Scomber australasicus***) and Chub Mackerel (***Scomber japonicus***) from 2014 to 2023. Panels (**a**,**b**) depict the changes in latitude and longitude centroids, along with their 95% confidence intervals (via bootstrap) and linear fits, for Blue Mackerel. Panels (**c**,**d**) present the corresponding data for Chub Mackerel.

To investigate the dynamics of the abundance centroid for Blue Mackerel and Chub Mackerel, we calculated their annual centroids and corresponding 95% bootstrap confidence intervals. Linear fitting was used to quantify the shifting trends of the centroids, with ordinary least squares (OLS) regression employed for statistical analysis. Results showed (Figure 7) that both species exhibited continuous northward shifts in their latitudinal centroids: Blue Mackerel moved from 40.61° N to 43.46° N, with a linear fitting slope (average annual rate) of 0.3062° per year (R^2^ = 0.820, *p* < 0.001). Residual analysis (Omnibus test, *p* = 0.773; Jarque–Bera test, *p* = 0.762) indicated that the data conformed to the assumptions of the linear model. In the longitudinal direction, Blue Mackerel’s centroid shifted eastward from 150.82° E to 157.10° E, with a fitting slope of 0.5736° per year (R^2^ = 0.583, *p* = 0.010), and residual analysis (Omnibus test, *p* = 0.481; Jarque–Bera test, *p* = 0.696) confirmed the validity of the linear assumption. For Chub Mackerel, its latitudinal centroid moved northward from 41.27° N to 43.72° N, with a linear fitting slope of 0.2708° per year (R^2^ = 0.732, *p* = 0.002), and residual analysis validated the rationality of the linear model. Its longitudinal centroid shifted eastward from 151.45° E to 157.00° E, with a fitting slope of 0.5125° per year (R^2^ = 0.533, *p* = 0.016), and residual analysis also supported the linear model. Further analysis of the spatial distance between the centroids of the two species revealed a continuous decrease. Specifically, Blue Mackerel had higher annual migration rates than Chub Mackerel in both latitude (0.31°/year vs. 0.27°/year) and longitude (0.57°/year vs. 0.51°/year). Over time, the faster northward and eastward shifts of Blue Mackerel narrowed the spatial gap with Chub Mackerel, leading to a gradual increase in the convergence of their distributions.

## 4. Discussion

### 4.1. Evaluation of ZOBIM Performance

As marine species that possess both critical ecological functions and significant fishery economic value, the precise management of mackerel populations is of great importance for maintaining marine ecosystem balance and supporting the sustainable development of fisheries [18]. However, the long-standing misidentification and aggregation of Blue Mackerel and Chub Mackerel in the fishery logbooks of various nations in the North Pacific have severely hindered the accurate assessment and scientific management of these two resources [13]. The ZOIBM, a hybrid model that integrates a continuous probability distribution with a discrete mechanism for extreme values, has demonstrated unique advantages in fields such as ecology and psychology [15,17,39], yet its application in marine fishery resource research has been hitherto unexplored. Unlike traditional approaches such as RF, which often handle only binary presence-absence data, the ZOIBM explicitly models both occurrence probability and proportional abundance, thereby capturing the zero-inflation and extreme values typical of fishery catch data. This allows for a more mechanistically consistent reconstruction of abundance patterns, reflecting the patchy and episodic aggregation behavior of Blue and Chub Mackerel. By filling this methodological gap, the study provides a practical framework for improving resource discrimination in mixed-catch fisheries and offers a data-driven basis for more precise spatial management.

This study introduces, for the first time, the ZOIBM into the analysis of marine biological resource abundance, reconstructing the abundance distribution patterns of Blue Mackerel and Chub Mackerel by integrating environmental factors and spatial autocorrelation. From a model performance perspective, the R-hat values for all parameters were well below the critical threshold of 1.05, indicating that the four MCMC chains had fully converged. This effectively avoided bias in parameter estimation and ensured the reliability and stability of the model parameters. The results of the Posterior Predictive Check further validated the model’s appropriateness, with a high degree of congruence between the simulated and observed data distributions. Through a multi-metric evaluation using block cross-validation, the model exhibited robust predictive capabilities: on the test set, the average RMSE was 0.305, the MAE was 0.298, and the average R^2^ reached 0.80, demonstrating that the model possesses good generalization ability and can make effective predictions on unseen data. However, despite efforts to ensure data accuracy—such as randomized sampling across multiple vessels/days with a minimum of 1000 individuals per event—uncertainty-related limitations persist. First, species ratios from finite samples have inherent sampling error, which was not explicitly incorporated into the ZOIBM, slightly affecting abundance reconstruction precision. Second, even with the region’s highest spatiotemporal resolution data, fine-scale discrepancies (e.g., gridded vs. in-situ environments) may still cause subtle spatiotemporal matching errors between fishery and environmental data.

### 4.2. Environmental Driving Mechanisms of Spatiotemporal Distributional Differences

As typical small pelagic commercial fish species, the population distributions of Chub Mackerel and Blue Mackerel are highly sensitive to changes in the marine environment [18]. However, due to the historical limitation of species misidentification in commercial fishery logbooks, traditional fisheries resource assessments have often analyzed the two as a single group or simplified the entire mackerel fishery into a single-species study of Chub Mackerel, owing to its dominant proportion in the catch. This approach has led to a lack of systematic investigation into the environmental drivers behind the distributional differences in abundance between the two mackerel species. Although isolated studies have revealed that Blue Mackerel prefers warmer, high-salinity environments compared to Chub Mackerel [9,19,40], the mechanisms of action for other key oceanographic factors have remained a research gap. By employing GAM, this study provides a detailed analysis of the environmental drivers of the differentiated abundance distributions of Blue and Chub Mackerel. The results indicate that SST, Chla, and VO are the core environmental factors driving the distributional differences between the two species (Figure 4).

SST exhibits significant species-specificity in its effect on the distribution of the two mackerels, making it the core factor driving their niche differentiation. The relative abundance of Chub Mackerel shows a unimodal response to SST, with its optimal temperature range concentrated at 17–19 °C, followed by a sharp decline. This reflects the strict dependence of Chub Mackerel on temperate, cooler water environments. In contrast, Blue Mackerel shows a monotonically increasing trend with SST, maintaining a high probability of occurrence even at the highest temperatures in the study area (approx. 23 °C), which show-cases the high-temperature adaptation preference of a subtropical species. This finding aligns with previous conclusions that Blue Mackerel prefers warmer waters [40]. As a subtropical species, Blue Mackerel may avoid direct competition with Chub Mackerel by expanding its distribution into warmer regions. Conversely, the strict adaptation of Chub Mackerel to lower temperatures limits its ability to expand toward lower latitudes. This, in turn, explains why the proportion of Blue Mackerel is low at high latitudes, while the proportion of Chub Mackerel is low at low latitudes.

The ecological effect of Chla, a core indicator of marine primary productivity [25], on the distribution of the two mackerel species shows significant interspecific differences, profoundly reflecting the divergence in their resource utilization strategies. The GAM smooth response curve (Figure 4B) shows that the response of Chub Mackerel to changes in Chla is flatter and more stable, with its partial effect consistently fluctuating around zero. This reflects a more “generalist” adaptation strategy to productivity environments driven by chlorophyll. That is, even with fluctuations in Chla, the distribution or survival of Chub Mackerel does not experience drastic oscillations, suggesting its resource utilization pattern does not depend on singular “pulses” of high productivity but rather maintains a relatively stable ecological performance across different Chla ranges. In contrast, the partial effect on Blue Mackerel exhibits a strong response of “first rapidly turning positive, then continuously climbing” as Chla increases. Specifically, its effect is negative when Chla is below 0.5 mg/m^3^, but it quickly surpasses the zero line in the 0.5–1.5 mg/m^3^ range, indicating it adapts to and benefits from rising productivity. This demonstrates that Blue Mackerel’s resource utilization strategy is more “specialized”—it is weakly adapted to low-Chla oligotrophic waters, but as productivity increases, its ecological advantage rapidly becomes prominent, showing a higher dependence on high-productivity warm water zones (such as nutrient-rich areas at lower latitudes). This clear difference in response is fundamentally a manifestation of niche complementarity. Chub Mackerel, with its “robust adaptation strategy,” maintains a stable distribution across various Chla ranges, allowing it to broadly utilize resources from different sea areas and reduce its dependence on any single environmental fluctuation. Blue Mackerel, on the other hand, employs a “high-productivity dependence strategy,” focusing on warm waters with high Chla to reduce interspecific competition by occupying specific high-productivity habitats. Through this mismatch of “generalist-specialist” resource utilization strategies, the two species achieve niche differentiation and coexistence, corroborating the classic ecological theory that species maintain community stability through strategic complementarity [41,42]. VO exhibits a non-linear inhibitory effect on the distribution of both mackerel species, but the response of Blue Mackerel is more pronounced, reflecting its high sensitivity to changes in north–south currents. This is because north–south currents (like the Oyashio and Kuroshio) indirectly shape the habitat by regulating temperature gradients and nutrient transport. Strong VO-induced cold-water upwelling or northward expansion of warm water can compress the cold-current boundary for Blue Mackerel’s expansion into higher latitudes (due to its sensitivity to low temperatures). Meanwhile, the core distribution area of Chub Mackerel (the mid-latitude transition zone) is more adaptable to current fluctuations, resulting in a flatter response [21,43,44].

UO, SSS, and MLD showed highly consistent responses in both mackerel species, contributing little to interspecific distribution differences. UO-driven east–west currents affect both species’ dispersal equally, failing to form niche barriers. MLD influences vertical mixing, but similar nutrient cycling requirements prevent distribution divergence. Though both respond to SSS with a unimodal trend, Blue Mackerel prefers slightly higher salinity (34.0‰ vs. 33.9‰ for Chub Mackerel). However, SSS fluctuates narrowly (30‰–35‰) in shared feeding grounds, making it a weak driver. This highlights that salinity’s niche-differentiating role depends on environmental heterogeneity—narrow fluctuations diminish its impact. Their distributions are dominated by temperature and chlorophyll-a, reflecting marine resource abundance as a synergistic result of multiple factors. Future studies in regions with larger salinity gradients could further validate salinity’s role (such as between Exclusive Economic Zones and the high seas).

### 4.3. Dynamics of Fishing Ground Centroids

Relying on fishery logbook data from 2014 to 2023, this study is the first to systematically reveal the synergistic evolution of the distribution patterns of Blue Mackerel and Chub Mackerel through a dynamic analysis of their latitudinal and longitudinal centroids (Figure 7). This provides critical evidence for understanding the response mechanisms of pelagic fish to climate change. The study found that over the decade, the latitudinal centroids of both mackerel species shifted significantly northward, a trend that is closely coupled with rising ocean temperatures driven by global warming [45]. As a subtropical species, Blue Mackerel exhibits adaptive northward expansion by tracking the poleward shift in warm water zones—mid-to-high latitude warming expanded its suitable thermal habitat, while extreme high temperatures compressed its low-latitude habitats, driving an overall northward push. In contrast, although Chub Mackerel is more sensitive to high temperatures, mid-latitude warming induced a poleward shift in its optimal thermal zone, triggering concurrent northward migration. More importantly, this “climate-warming-induced shift in the optimal thermal zone” not only redefines the habitat boundaries for both species but also alters the distribution of productivity in high-latitude waters by reshaping phytoplankton community structures (e.g., changes in dominant species, shifts in phenology) and nutrient transport patterns. This, in turn, drives the mackerel to migrate toward these “zones of productivity reorganization,” demonstrating a synergistic trend in the distributional responses of the two species [46,47]. Furthermore, the distribution centroids of both mackerel species also shifted significantly eastward. This is likely influenced by the dual impacts of coastal overfishing and climate change, which have caused the biomass of key forage resources, such as sardines, to shift eastward [20,48]. To meet their feeding demands, the mackerels have adaptively migrated eastward, tracking the movement of their prey into more easterly waters. The dynamics of the distribution centroids (Figure 7) revealed that the spatial distance between Blue Mackerel and Chub Mackerel consistently decreased during the study period, showing a significant linear convergence trend. This pattern indicates that their core habitats have become increasingly overlapping, suggesting that the two species are progressively occupying shared ecological space. From an ecological perspective, the enhanced overlap of habitats and forage resources raises the possibility of intensified interspecific competition. Given that Blue Mackerel and Chub Mackerel share highly similar ecological niches—both being small pelagic species that often co-occur and primarily feed on copepods and juvenile sardines—spatial convergence provides indirect evidence of potential competitive interactions. However, direct empirical evidence of competition between the two species remains lacking, making it difficult to determine whether distributional overlap necessarily translates into resource competition and how this might affect their population dynamics. Future research should therefore integrate stomach content analysis, stable isotope techniques, and population dynamic models with explicit interspecific competition terms in order to quantify trophic niche overlap and assess the demographic consequences of competition. Such efforts will provide a stronger scientific basis for the coordinated management of these shared fishery resources [20,49,50].

### 4.4. Management Recommendations

Based on the distribution characteristics and environmental driving mechanisms of the two mackerel species revealed in this study, the following comprehensive recommendations are proposed to achieve sustainable management of the North Pacific mackerel fishery. To address the issue of mixed-species reporting in catch data, a two-pronged solution combining “technical calibration and skill enhancement” is required. On one hand, a standardized catch data calibration system should be established based on the Zero-Inflated Ordered Biomass Model (ZOIBM) proposed in this study. This system leverages the species proportion characteristics output by the ZOIBM to retroactively correct historically misreported species data, while also integrating a model calibration module into real-time catch recording processes to verify and revise data submitted by fishermen. This breaks through the limitations of traditional real-time identification from a technical perspective and aligns with the core objective of this study—”overcoming data deficiencies using advanced models”. On the other hand, simultaneous efforts should be made to strengthen species identification training for fishermen and promote standardized, detailed logbook recording. By improving the basic identification capabilities of frontline data collectors, errors in raw data (including mixed-species reporting) are reduced, providing reliable initial data support for model calibration. The synergy between these two approaches will significantly enhance the accuracy of catch data, laying a precise baseline for quota allocation by the North Pacific Fisheries Commission (NPFC). Concurrently, considering the critical influence of environmental factors such as SST on the distribution of both mackerel species, marine environmental changes should be integrated into the resource assessment framework, and dynamic management plans based on environmental indicators should be established. Furthermore, given the northward and eastward shifting trend of the species’ distribution centroids, it is essential to enhance transnational cooperative monitoring to jointly maintain the ecological balance of newly expanded high-latitude habitats. Through collaborative scientific research and management measures, regional fishing activities should be coordinated to ensure that both mackerel resources can be utilized sustainably, especially in the context of their increasing niche overlap.

## 5. Conclusions

This study is the first to introduce a ZOIBM, in conjunction with a GAM, to conduct an in-depth analysis of the relative abundance distribution and environmental differences in Blue Mackerel and Chub Mackerel on the high seas of the North Pacific. The results demonstrate that the ZOIBM can effectively quantify the proportional composition of the two mackerel species in mixed catches and accurately delineate their spatiotemporal distribution patterns. Significant differences exist in the spatial distributions of Blue and Chub Mackerel, with SST, Chla, and VO identified as the core environmental factors driving these distributional differences. Between 2014 and 2023, the distribution centroids of both species showed significant northward and eastward shifts, with their spatial overlap continuously increasing. This research provides a new methodological reference for the fine-scale assessment of co-occurring fish resources and offers a scientific basis for the sustainable management of the North Pacific mackerel fishery.

## Figures and Tables

**Table 1:** Source of environmental data.

Environmental Variable	Data Source	Initial Temporal Resolution	Initial Spatial Resolution
Sea Surface Temperature (/°C)	GOPR	Daily	0.083° × 0.083°
Chlorophyll-a Concentration (mg/m^3^)	GOBR	Daily	0.25° × 0.25°
Sea Surface Salinity (‰)	GOPR	Daily	0.083° × 0.083°
Sea Surface Height (m)	GOPR	Daily	0.083° × 0.083°
Eastward sea water velocity (m/s)	GOPR	Daily	0.083° × 0.083°
Northward sea water velocity (m/s)	GOPR	Daily	0.083° × 0.083°
Mixed Layer Depth (m)	GOPR	Daily	0.083° × 0.083°

Notes: GOPR: Global Ocean Physics Reanalysis (available at https://data.marine.copernicus.eu, accessed on 21 January 2025). GOBR: Global Ocean Biogeochemical Reanalysis (available at https://data.marine.copernicus.eu, accessed on 21 January 2025).

**Table 2:** Gelman-Rubin Statistics (R-hat) for Model Parameters.

Number	Parameter	R-Hat Value
1	b_Intercept	1.0010054
2	b_phi_Intercept	0.9999693
3	b_zoi_Intercept	1.0018218
4	b_coi_Intercept	1.0004148
5	Spatial Random Effects (mean ± SD)	1.0013 ± 0.001

Notes: b_Intercept: Intercept of the continuous Beta distribution component. b_phi_Intercept: Intercept for the precision parameter (φ) of the Beta distribution, controlling distribution spread. b_zoi_Intercept: Intercept for the zero-inflation (ZOI) component (probability of exact zeros). b_coi_Intercept: Intercept for the one-inflation (COI) component (probability of exact ones).

**Table 3:** GAM Parameters for the Responses of Blue Mackerel and Chub Mackerel to Key Environmental Factors.

Variable	Species	Effective Degrees of Freedom (edf)	Reference Degrees of Freedom (Ref.df)	*p*-Value
Chla	Blue Mackerel	8.921019	8.998026	<0.001
	Chub Mackerel	8.926323	8.99829	<0.001
MLD	Blue Mackerel	3.687771	4.540656	<0.001
	Chub Mackerel	4.685233	5.637041	<0.001
SSS	Blue Mackerel	7.26042	8.215804	<0.001
	Chub Mackerel	7.137682	8.122104	<0.001
SST	Blue Mackerel	8.38792	8.890325	<0.001
	Chub Mackerel	8.383943	8.888969	<0.001
UO	Blue Mackerel	7.518612	8.366241	<0.001
	Chub Mackerel	7.579307	8.407768	<0.001
VO	Blue Mackerel	8.351075	8.860459	<0.001
	Chub Mackerel	8.312896	8.84484	<0.001

## Data Availability

Data will be made available on request.
